# Anti‐HER2 Immunoliposomes: Antitumor Efficacy Attributable to Targeted Delivery of Anthraquinone‐Fused Enediyne

**DOI:** 10.1002/advs.202307865

**Published:** 2024-02-14

**Authors:** Xueqiong Feng, Zhongqing Wen, Xiangcheng Zhu, Xiaohui Yan, Yanwen Duan, Yong Huang

**Affiliations:** ^1^ Xiangya International Academy of Translational Medicine Central South University Changsha Hunan 410013 China; ^2^ Hunan Engineering Research Center of Combinatorial Biosynthesis and Natural Product Drug Discover Changsha Hunan 410011 China; ^3^ State Key Laboratory of Component‐based Chinese Medicine Tianjin University of Traditional Chinese Medicine Tianjin 301617 China; ^4^ National Engineering Research Center of Combinatorial Biosynthesis for Drug Discovery Changsha Hunan 410011 China; ^5^ Institute of Health and Medicine Hefei Comprehensive National Science Center Hefei Anhui 230093 China

**Keywords:** anthraquinone‐fused enediyne, breast cancer, immunoliposomes, tiancimycin A, trastuzumab

## Abstract

Although natural products are essential sources of small‐molecule antitumor drugs, some can exert substantial toxicities, limiting their clinical utility. Anthraquinone‐fused enediyne natural products are remarkably potent antitumor drug candidates, and uncialamycin and tiancimycin (TNM) A are under development as antibody‐drug conjugates. Herein, a novel drug delivery system is introduced for TNM A using anti‐human epidermal growth factor receptor 2 (HER2) immunoliposomes (ILs). Trastuzumab‐coated TNM A‐loaded ILs (HER2‐TNM A‐ILs) is engineered with an average particle size of 182.8 ± 2.1 nm and a zeta potential of 1.75 ± 0.12 mV. Compared with liposomes lacking trastuzumab, HER2‐TNM A‐ILs exhibited selective toxicity against HER2‐positive KPL‐4 and SKBR3 cells. Coumarin‐6, a fluorescent TNM A surrogate, is encapsulated within anti‐HER2 ILs; the resultant ILs have enhanced cellular uptake in KPL‐4 and SKBR3 cells when compared with control liposomes. Furthermore, ILs loaded with more Cy5.5 accumulated in KPL‐4 mouse tumors. A single HER2‐TNM A‐IL dose (0.02 mg kg^−1^) suppressed the growth of HER2‐positive KPL‐4 mouse tumors without apparent toxicity. This study not only provides a straightforward method for the effective delivery of TNM A against HER2‐positive breast tumors but also underscores the potential of IL‐based drug delivery systems when employing highly potent cytotoxins as payloads.

## Graphical abstract

A novel immunoliposome drug delivery system comprised of trastuzumab and enediyne natural product tiancimycin A is prepared and evaluated against HER2‐positive breast tumor mouse model, revealing that a single dose of the immunoliposome (0.02 mg kg^−1^) significantly suppressed the tumor growth without obvious toxicity.

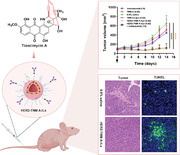

## 1 Introduction

In an age marked by multispecific drug innovation, novel modalities such as antibody‐drug conjugates (ADCs), molecular glues, and proteolysis‐targeting chimeras have emerged as pivotal approaches for addressing diseases that were hitherto refractory to conventional therapies.^[^ ^1^ ^]^ Notably, trastuzumab deruxtecan (T‐DXd, formerly DS‐8201) serves as an illustrative example of ADCs, using trastuzumab, a recombinant humanized monoclonal antibody targeting the extracellular domain of human epidermal growth factor receptor 2 (HER2), in conjunction with a potent DNA topoisomerase I inhibitor.^[^ ^2^ ^]^ T‐DXd has demonstrated anticancer efficacy in HER2‐positive metastatic breast cancer and a spectrum of solid malignancies, including previously treated HER2‐mutant non‐small‐cell lung cancer and HER2‐low tumors.^[^ ^3^ ^]^ These facts not only underscore the success of HER2‐targeted therapies but also illuminate the vast potential of ADCs, with 14 currently holding approval status and over 140 undergoing diverse phases of clinical trials.^[^ ^4^ ^]^ Nonetheless, considerable challenges hinder the development of ADCs, as reflected by the discontinuation of more than 100 ADC programs. Recently, Colombo and Rich examined ten approved ADCs, positing that existing ADCs failed to significantly expand the therapeutic window of their respective payloads.^[^ ^5^ ^]^

**Scheme 1:**
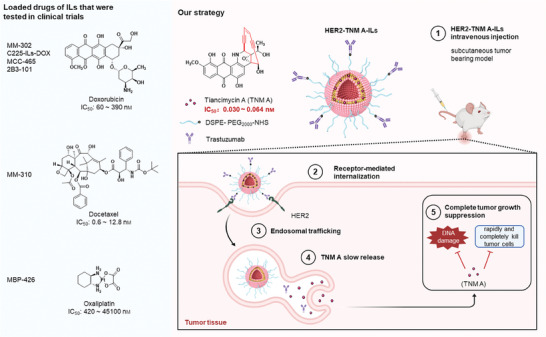
Schematic illustration of TNM A‐loaded immunoliposomes and antitumor therapy. HER2, human epidermal growth factor receptor 2; ILs, immunoliposomes; TNM A, tiancimycin A.

Drug delivery systems can effectively maintain the concentrations of certain therapeutics within a therapeutic window for prolonged periods.^[^ ^6^ ^]^ The development of appropriate drug delivery systems would increase tumor exposure to anticancer treatments while simultaneously minimizing systemic exposure.^[^ ^7^ ^]^ Moreover, the enhanced permeability and retention (EPR) effect, a phenomenon harnessed by biomaterials such as liposomes, micelles, and inorganic carriers, facilitates the efficient delivery of diverse payloads to tumor sites.^[^ ^8^ ^]^ Among these biomaterials, immunoliposomes (ILs) represent a class of materials designed to actively target solid tumors through the surface attachment of monoclonal or polyclonal antibodies.^[^ ^9^ ^]^ For example, lung‐targeting ILs have been used to enhance drug delivery to the lungs using the monoclonal antibody 273‐34A coupled with palmitic acid.^[^ ^10^ ^]^ These antibodies serve as guiding mechanisms to direct ILs to their intended destinations, with payloads enclosed either internally or within the lipid bilayers of ILs (**Scheme** **1**).^[^ ^11^ ^]^ For instance, PEGylated ILs carrying the anti‐HER2 antibody trastuzumab, such as MM‐302, have shown promise in the treatment of HER2‐overexpressing breast cancer by encapsulating the antitumor agent doxorubicin.^[^ ^12^ ^]^ It is well‐established that PEGylation can extend the circulation time of ILs in the bloodstream, while trastuzumab enhances the therapeutic index of doxorubicin by enabling a more selective delivery.^[^ ^13^ ^]^ Other ILs employ distinct antibodies (e.g., C225‐ILs‐DOX, MCC‐465, and 2B3‐101) to achieve targeted doxorubicin delivery, while ILs containing microtubule inhibitors, such as docetaxel, or DNA alkylation agents, such as oxaliplatin (e.g., MM‐310 or MBP‐426), are also in various stages of development.^[^ ^11^, ^14^ ^]^ Recent advances in IL technology, such as the utilization of superstealth or multifunctional ILs, site‐selective modifications, and encapsulation of diverse toxins, offer promising insights for accelerating IL translation.^[^ ^15^ ^]^

Despite these developments, the formulated ILs are yet to receive market approval, and this can be attributed to the following factors: 1) the immunosuppressive tumor microenvironment can compromise the efficacy of antibody‐dependent cell‐mediated cytotoxicity and 2) antibody surface density may influence the liposome binding affinity, subsequent cellular uptake, and payload release.^[^ ^11^ ^]^ Furthermore, the payloads in ILs currently undergoing clinical trials primarily comprise well‐established chemotherapeutic drugs such as doxorubicin, docetaxel, and oxaliplatin, with half‐maximal inhibitory concentrations (IC_50_) largely falling within the nanomolar to micromolar range.^[^ ^12^, ^14^ ^]^ In light of the successful development of approved ADCs, the inclusion of highly potent toxins, such as calicheamicin and camptothecin derivatives, may be instrumental in enhancing IL efficacy.^[^ ^16^ ^]^ Notably, a recent setback in a phase II trial assessing anti‐epidermal growth factor receptor 1‐ILs loaded with doxorubicin as a first‐line therapy for advanced triple‐negative breast cancer further underscores the formidable challenges facing IL‐based antitumor therapy.^[^ ^17^ ^]^

Enediyne natural products represent a distinctive family of exceptionally potent antitumor small‐molecule drugs characterized by unique molecular structures and novel mechanisms of action.^[^ ^18^ ^]^ Owing to their inherent toxicity toward both tumor and normal cells,^[^ ^19^ ^]^ the clinical utilization of enediynes necessitates the use of conjugated carriers, such as antibodies, to enhance their targeting specificity. For example, calicheamicin serves as the payload in Mylotarg (gemtuzumab ozogamicin) and Besponsa (inotuzumab ozogamicin), approved for treating acute myeloma leukemia and B‐cell precursor acute lymphoblastic leukemia, respectively.^[^ ^20^ ^]^ Notably, Mylotarg has been instrumental in advancing the field of multispecific drug innovation since the year 2000.^[^ ^1^, ^21^ ^]^ In Japan, SMANCS, a copolymer comprising maleimide and enediyne neocarzinostatin, has been used to treat hepatomas since 1994.^[^ ^22^ ^]^

One distinct subset of enediynes, specifically anthraquinone‐fused enediynes, includes dynemicin A, uncialamycin, tiancimycin (TNM) A, and yangpumicins, which are distinguished by the fusion of anthraquinones with enediyne carbocycles.^[^ ^23^ ^]^ This structural feature enables their interaction with double‐stranded DNA, followed by DNA double strand cleavage upon reductive activation.^[^ ^18^ ^]^ In preclinical investigations, uncialamycin‐based ADCs have demonstrated robust antitumor effects in mouse models.^[^ ^24^ ^]^ However, the limited delivery of ADCs to human tumors may hinder the clinical translation of these promising findings, as observed with previously marketed ADCs, with the remainder contributing to undesired toxicity.^[^ ^5^ ^]^ The interplay between ADC tumor targeting, prolonged payload release in the circulation, and the inherent action of the targeting antibody converge to enhance the efficacy of ADCs.^[^ ^25^ ^]^

Accordingly, we propose that encapsulating highly toxic drugs or drug leads within biomaterials, specifically ILs, may yield superior therapeutic outcomes to those of less potent payloads. Furthermore, the IL‐based targeted delivery of these potent toxins could mitigate their off‐target effects and reduce their toxicity in normal cells. We have explored the encapsulation of TNM A within liposomes and assessed its antitumor activity in vivo, in which TNM A‐liposome treatment group exhibited better tumor inhibition than the control group.^[^ ^19b^ ^]^ In the current study, we selected TNM A‐encapsulated in trastuzumab‐based ILs as a prototype to establish the design, preparation, and characterization of ILs loaded with highly potent toxins (Scheme 1). This study encompasses the synthesis of HER2‐targeting, TNM A‐loaded ILs, along with the characterization of their particle size, zeta potential, morphology, and drug release kinetics. Additionally, the cytotoxicity, cellular uptake, and antitumor effects of TNM A‐loaded ILs were evaluated comprehensively. Our investigations unequivocally demonstrate that ILs represent a highly effective platform for the targeted delivery of TNM A, presenting a promising avenue for improving therapeutic outcomes.

## 2 Results and Discussion

### 2.1 Preparation and Characterization of Immunoliposome Drug Delivery Systems

The drug‐to‐antibody dosing ratio can profoundly influence the cytotoxicity of ILs. For instance, MM‐302 ILs have been engineered to encapsulate ≈20000 molecules of doxorubicin within their cores in conjunction with 45 single‐chain anti‐HER2 antibodies.^[^ ^12a^ ^]^ TNM A is a potent antitumor antibiotic exhibiting efficacy within the picomolar to single‐digit nanomolar range.^[^ ^19a^ ^]^ Unlike widely used antitumor agents such as doxorubicin, with IC_50_ values ranging from 60 to 390 nm against a broad spectrum of cancer cell lines, we postulated that TNM A‐encapsulated ILs may not necessitate large loading doses to achieve enhanced antitumor effects. To develop HER2‐TNM A‐ILs, TNM A‐encapsulated liposomes were first generated using the ethanol evaporation method, with phosphatidylcholine (PC), cholesterol, DSPE‐PEG_2000_, and DSPE‐PEG_2000_‐NHS used in a molar ratio of 56:39:5:1 (Figure S1, Suppporting information). Subsequently, TNM A was added and mixed thoroughly, which resulted in liposome formation. Finally, the prepared liposomes were incubated with dialyzed trastuzumab at a molar ratio of 1:50 to facilitate the formation of HER2‐TNM A‐ILs, with trastuzumab attached to the liposome surface via an amide linkage. Notably, no aromatization of TNM A was observed during liposome formulation (Figure S2, Supporting Information).

A series of HER2‐TNM A‐ILs with varying ratios of trastuzumab to TNM A (1:0.2, 1:1, 1:5, 1:10, and 1:30) was prepared (**Table** **1**; and Figure S1, Supporting Information), followed by the assessment of cytotoxicity using the HER2‐positive human breast cancer cell line KPL‐4 and the normal human colon mucosal epithelial cell line NCM‐460. Cytotoxicity was assessed over 8 and 48 h using the Cell Counting Kit‐8 assay. In KPL‐4 cells, the IC_50_ values varied between 5.54 and 49.52 nm after 8 h of incubation. Notably, ILs with the lowest TNM A load (1:0.2) exhibited the most potent cytotoxicity, with an IC_50_ of 5.54 nm against KPL‐4 cells. Furthermore, this IL category exerted the lowest toxicity toward normal NCM‐460 cells.

**Table 1:** Cytotoxicity evaluation of TNM A‐loaded ILs with different antibody/drug ratios.

Material Composition	Molar Ratio	IC_50_ (nm)			
		KPL‐4 (8 h)	KPL‐4 (48 h)	NCM‐460 (8 h)	NCM‐460 (48 h)
PC / CHOL / DSPE‐PEG_2000_ / DSPE‐PEG_2000_‐NHS / Trastuzumab / TNM A	56 : 39 : 5 : 0 : 0 : 0.2	12.22	0.93	41.25	4.17
	56 : 39 : 5 : 1 : 0.02 : 0.004	5.54	0.54	37.80	5.01
	56 : 39 : 5 : 1 : 0.02 : 0.02	13.16	2.26	45.28	3.23
	56 : 39 : 5 : 1 : 0.02 : 0.1	49.52	2.41	63.28	3.63
	56 : 39 : 5 : 1 : 0.02 : 0.2	11.06	1.43	49.43	3.07
	56 : 39 : 5 : 1 : 0.02 : 0.6	12.22	1.09	40.06	3.18

TNM A‐loaded ILs, tiancimycin A‐loaded immunoliposomes.

**Figure 1:**
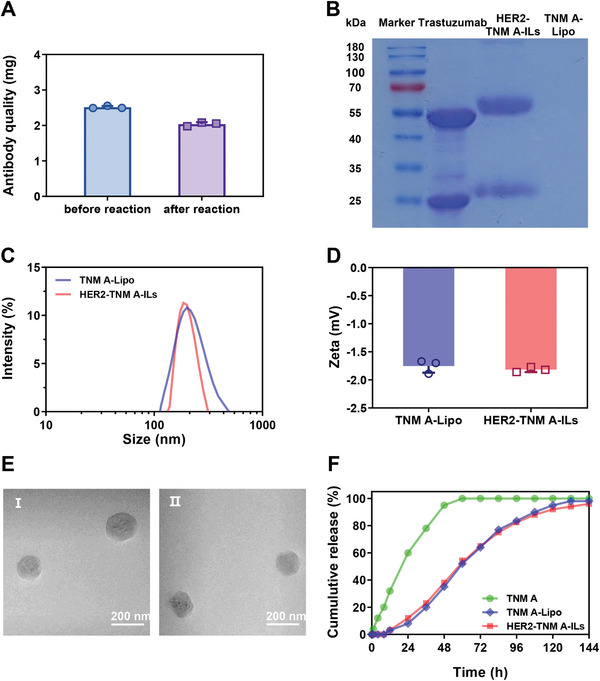
Characterization of TNM A‐Lipo and HER2‐TNM A‐ILs. A) Trastuzumab conjugation percentage of HER2‐TNM A‐ILs. B) SDS‐PAGE analysis of the integrity of trastuzumab in HER2‐TNM A‐ILs. C) Hydrodynamic sizes of TNM A‐Lipo and HER2‐TNM A‐ILs determined by dynamic light scattering. D) Zeta potentials of TNM A‐Lipo and HER2‐TNM A‐ILs determined using dynamic light scattering (*n* = 3). E) TEM photographs of TNM A‐Lipo (I) and HER2‐TNM A‐ILs (II). F) The release profile of free TNM A, TNM A‐Lipo, and HER2‐TNM A‐ILs in PBS (pH 7.4, 0.5% SDS) (*n* = 3). Error bars are representative of at least three replicates ± standard deviation (SD). HER2‐TNM A‐ILs, anti‐human epidermal growth factor receptor 2 (trastuzumab)‐coated tiancimycin A‐loaded immunoliposomes; IL, immunoliposomes; PAGE, polyacrylamide gel electrophoresis; PBS, phosphate‐buffered saline; SDS, sodium dodecyl sulfate; TEM, transmission electron microscopy; TNM A, tiancimycin A.

A similar trend was evident in both cell types after 48 h of treatment. Increasing the molar ratio of TNM A to the antibody reduced the cytotoxicity and selectivity of the ILs. Accordingly, we selected a TNM A‐to‐antibody feeding ratio of 0.004:0.02 for subsequent experiments. The amount of trastuzumab covalently conjugated to liposomes was determined indirectly by subtracting the unreacted monoclonal antibody from the total amount of trastuzumab applied, yielding an average coupling efficiency of 81% (**Figure** **1**A). To further verify the presence and integrity of trastuzumab in HER2‐TNM A‐ILs, sodium dodecyl sulfate‐polyacrylamide gel electrophoresis (SDS‐PAGE) analysis was performed, revealing the presence of heavy chains at 50 kDa and light chains at 25 kDa; these were slightly larger than those of free trastuzumab, owing to the presence of DSPE‐PEG_2000_ (Figure 1B).

The average diameters of TNM A‐Lipo and HER2‐TNM A‐ILs were 182.8 ± 2.1 and 202.8 ± 1.5 nm, respectively. Upon trastuzumab attachment, the particle size of the HER2‐TNM A‐ILs decreased by ≈20 nm, suggesting the invagination of the phospholipid bilayer (Figure 1C). The zeta potential of TNM A‐Lipo was −1.75 ± 0.12 mV, whereas the liposome formulation exhibited a slightly lower surface negative charge (Figure 1D). Transmission electron microscopy (TEM) images further confirmed the spherical vesicular nature of both TNM A‐Lipo and HER2‐TNM A‐ILs (Figure 1E). Using a two‐chamber model, the release kinetics of HER2‐TNM A‐ILs were assessed and compared with those of TNM A‐Lipo. Both TNM A‐Lipo and HER2‐TNM A‐ILs displayed no burst release, and < 40% of TNM A was released over 48 h under physiological conditions (pH 7.4, 37 °C, 0.5% SDS), in line with our previous findings.^[^ ^19b^ ^]^ This observation indicated that HER2‐TNM A‐ILs exhibited minimal drug leakage and a sustained release profile (Figure 1F). Nanoparticle flow cytometry was used to measure the particle concentration of HER2‐TNM A‐ILs, showing ≈2.33 ± 0.23 × 10^12^ particles mL^−1^ (Figure S3, Supporting Information). Based on the concentrations of the encapsulated TNM A and modified trastuzumab, each IL contained an average of ≈190 TNM A and ≈830 trastuzumab molecules. Consequently, the prepared ILs represent an effective nanoplatform to achieve the efficient and stable encapsulation of enediyne antitumor agents such as TNM A.

### 2.2 Targeted Delivery of HER2‐TNM A‐ILs in HER2‐Overexpressing Breast Tumor Cells

**Figure 2:**
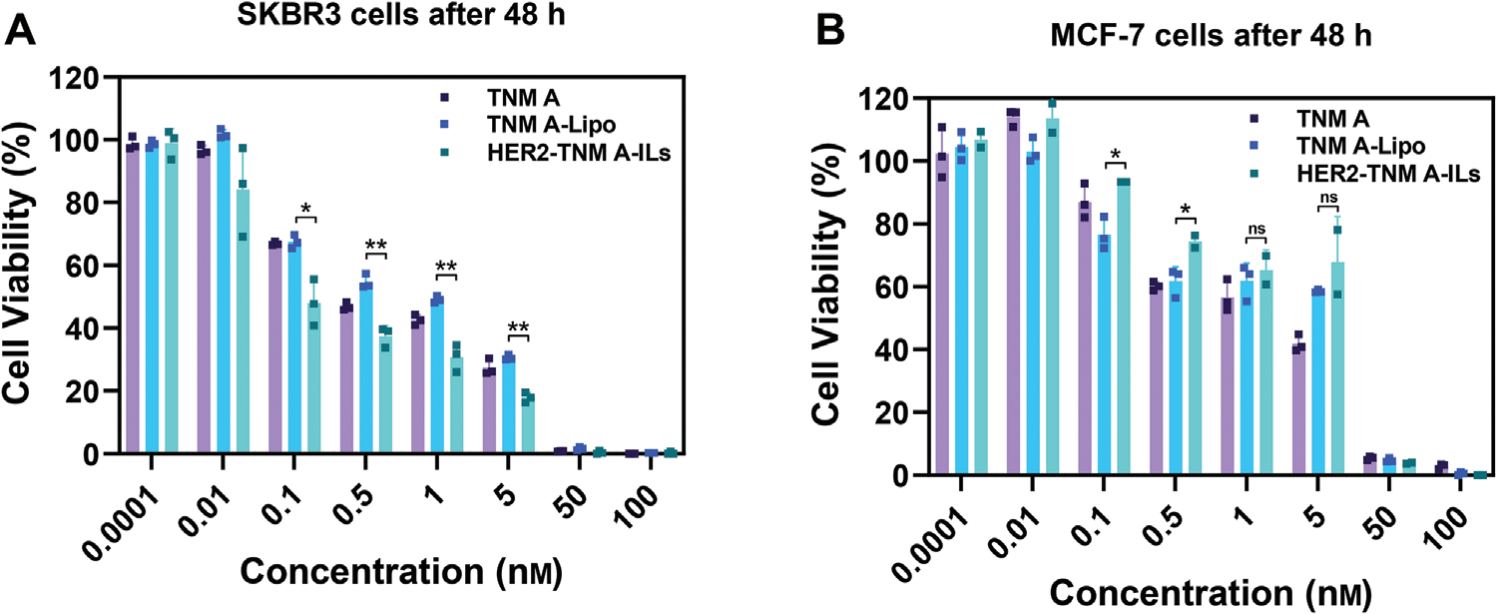
In vitro cell cytotoxicity. A) In vitro cytotoxicities of TNM A, TNM A‐Lipo, and HER2‐TNM A‐ILs against SKBR3 cells (*n* = 3). B) In vitro cytotoxicities of TNM A, TNM A‐Lipo, and HER2‐TNM A‐ILs against MCF‐7 cells (*n* = 3). Data are presented as the mean ± SD, One‐way ANOVA test (A,B), (*) < 0.05, (**) < 0.01 and not significant (ns). HER2‐TNM A‐ILs, anti‐human epidermal growth factor receptor 2 (trastuzumab)‐coated tiancimycin A‐loaded immunoliposomes; IL, immunoliposomes; TNM A, tiancimycin A.

We next examined the cytotoxicity and selectivity of HER2‐TNM A‐ILs using SKBR3 and MCF‐7 cells. SKBR3 is a human breast cancer cell line characterized by *HER2* gene overexpression, whereas MCF‐7 cells lack *HER2* overexpression.^[^ ^26^ ^]^ The cytotoxicity of blank HER2‐ILs was evaluated in SKBR3 and MCF‐7 cells. Cells treated with blank HER2‐ILs at varying concentrations (0.01−1.0 mg mL^−1^) displayed >90% cell viability (Figure S4, Supporting Information), indicating that the liposome carrier had minimal cytotoxicity. In SKBR3 cells, HER2‐TNM A‐ILs exerted remarkable cytotoxicity (IC_50_ = 0.24 nm); this IC_50_ value was 3‐fold lower than that of non‐targeted TNM A‐Lipo (0.73 nm) and 2‐fold lower than that of free TNM A (0.51 nm) (**Figure** **2**A; Figure S5, Supporting Information). Conversely, in MCF‐7 cells, HER2‐TNM A‐ILs displayed comparable cytotoxicity with an IC_50_ of 1.54 nm, which did not differ significantly from non‐targeted Lip‐TNM A (1.89 nm) or free TNM A (1.73 nm) (Figure 2B; Figure S5, Supporting Information). The substantial discrepancy in cytotoxicity between HER2‐TNM A‐ILs and TNM A‐Lipo in SKBR3 cells underscores the superior targeting efficiency of these ILs toward HER2‐positive breast cancer cell lines.

### 2.3 Cellular Internalization

The therapeutic efficacy of ILs largely depends on their internalization by target cells. Accordingly, we next evaluated the cellular uptake of trastuzumab‐bearing ILs using inverted fluorescence microscopy and flow cytometry in HER2‐expressing KPL‐4 and SKBR3 cells. Both cell types are commonly used in preclinical studies on breast cancer. Coumarin‐6 (Cou‐6), a green fluorescent dye, is widely used for assessing the uptake of nanoparticles by tumor cells. Liposome‐encapsulated Cou‐6 (Cou‐6‐Lipo) and IL‐encapsulated Cou‐6 (HER2‐Cou‐6‐ILs) were prepared under the same conditions used to prepare the TNM A‐encapsulated liposomes (Figure S6, Supporting Information).

**Figure 3:**
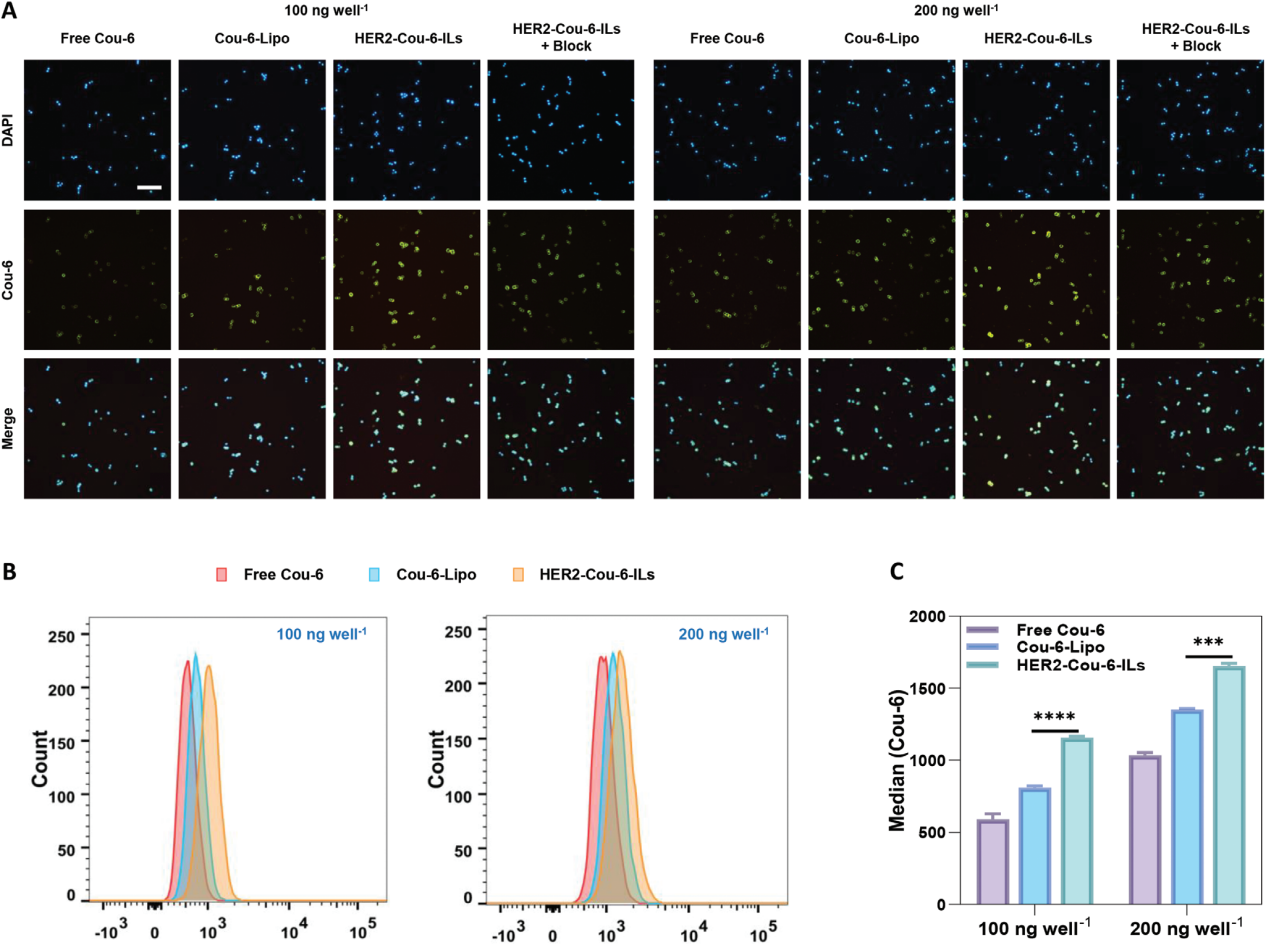
Cell uptake of free Cou‐6, Cou‐6‐Lipo, and HER2‐Cou‐6‐ILs as TNM A surrogates. A) Cellular uptake assessment of free Cou‐6, Cou‐6‐Lipo, and HER2‐ Cou‐6‐ILs in KPL‐4 cells at 100 and 200 ng well^−1^ using inversion fluorescence microscopy. Green: Cou‐6; Blue: DAPI. B) Cellular uptake of Cou‐6, Cou‐6‐Lipo, and HER2‐Cou‐6‐ILs in KPL‐4 cells using flow cytometry. C) Median fluorescence intensity of free Cou‐6, Cou‐6‐Lipo, and HER2‐Cou‐6‐ILs in KPL‐4 cells (*n* = 3). Data are presented as the mean ± SD, One‐way ANOVA test (C), (*) < 0.05, (**) < 0.01, (***) < 0.001, and (****) < 0.0001. Cou‐6‐Lipo, coumarin‐6‐Lipo; HER2‐Cou‐6‐ILs, anti‐human epidermal growth factor receptor 2 (trastuzumab)‐coated coumarin‐6‐loaded immunoliposomes; IL, immunoliposomes; TNM A, tiancimycin A.

KPL‐4 and SKBR3 cells were incubated with Cou‐6, Cou‐6‐Lipo, or HER2‐Cou‐6‐ILs for 2 h at 100 or 200 ng well^−1^, respectively. During the 2‐h incubation, we noted increasing fluorescence accumulation in KPL‐4 and SKBR3 cells (**Figure** **3**; Figure S7, Supporting Information), revealing dose‐dependent uptake. HER2‐Cou‐6‐ILs entered both cell types more efficiently than Cou‐6‐Lipo, indicating that the presence of trastuzumab in ILs facilitated their internalization. We further confirmed the HER2‐mediated active uptake using a receptor saturation inhibition assay. After saturation with trastuzumab (10 µM), the uptake of HER2‐Cou‐6‐ILs was dramatically reduced in KPL‐4 and SKBR3 cells (Figure 3A; Figure S7, Supporting Information). In KPL‐4 cells, treatment with HER2‐Cou‐6‐ILs increased Cou‐6 levels when compared with treatment with Cou‐6‐Lipo and free Cou‐6, as determined by flow cytometry analysis (Figure 3B). Flow cytometry analysis revealed that HER2‐Cou‐6‐ILs were internalized into KPL‐4 cells more efficiently than Cou‐6‐Lipo and free Cou‐6 (by 1.4‐fold and 1.9‐fold, respectively; Figure 3C). Based on the presence of HER2 receptors on KPL‐4 and SKBR3 cell surfaces, it can be confirmed that the incorporation of trastuzumab into liposomes successfully enhanced the internalization of HER2‐Cou‐6‐ILs.

### 2.4 In Vivo Imaging

**Figure 4:**
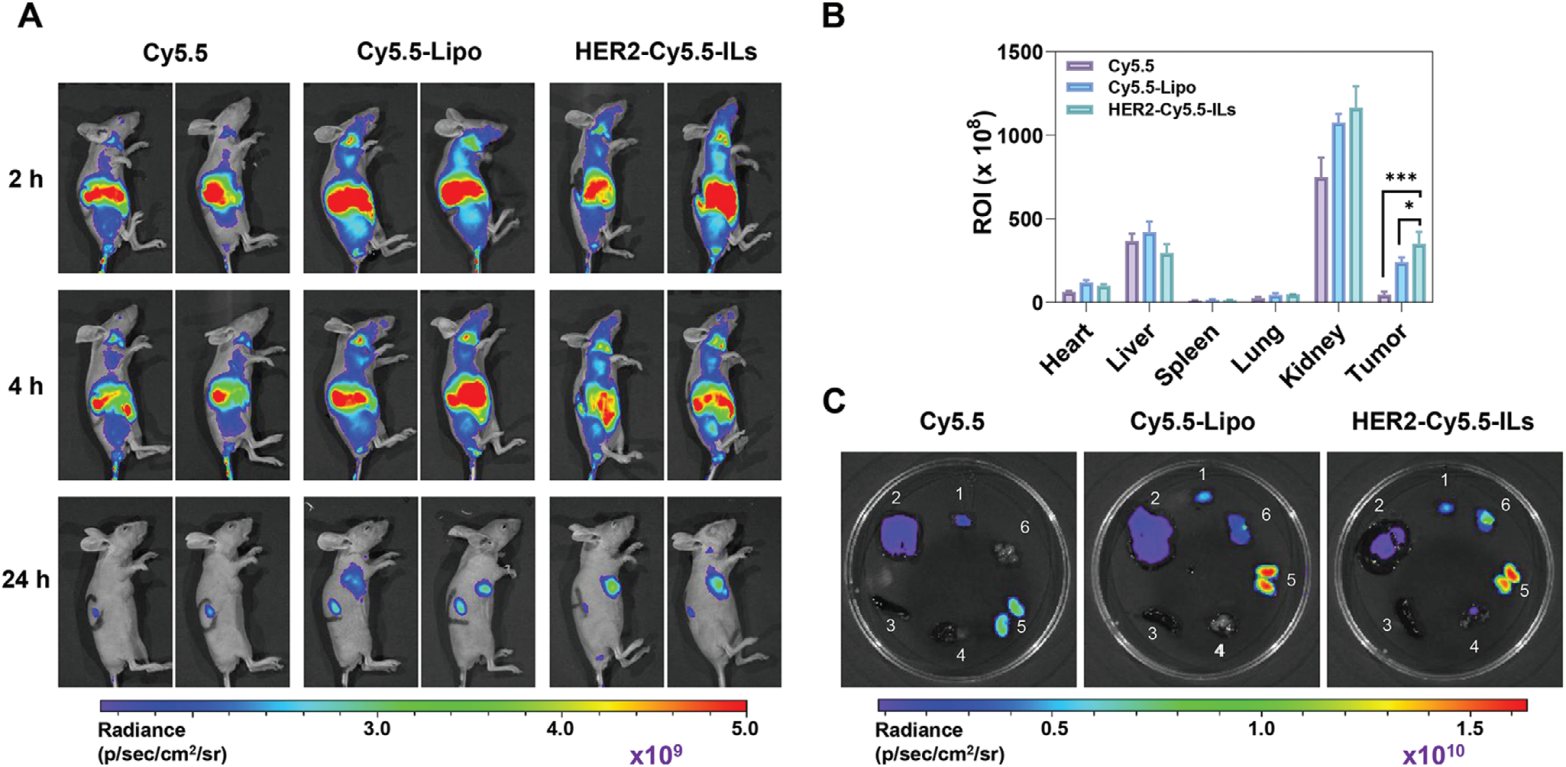
In vivo biodistribution assays of Cy5.5, Cy5.5‐Lipo, and HER2‐Cy5.5‐ILs. A) In vivo fluorescence imaging of Cy5.5, Cy5.5‐Lipo, and HER2‐Cy5.5‐ILs injected intravenously into KPL‐4 tumor‐bearing nude mice. B) Quantitative fluorescence intensities of main organs and tumors (*n* = 4). C) Fluorescence images of isolated (1) heart, (2) liver, (3) spleen, (4) lung, (5) kidney, and (6) tumor tissue 24 h post‐injection. Data are presented as the mean ± SD, One‐way ANOVA test (B), (*) < 0.05, (**) < 0.01, and (***) < 0.001. IL, immunoliposomes; HER2, human epidermal growth factor receptor 2.

We next evaluated the active targeting capability of trastuzumab‐based ILs in vivo. Accordingly, free Cy5.5, Cy5.5‐labeled liposomes (Cy5.5‐Lipo), and immunoliposomes (HER2‐Cy5.5‐ILs) were intravenously injected into mice with KPL‐4 tumors, and the in vivo fluorescence intensity was monitored for 24 h. Only liposomes and ILs exhibited substantial in vivo fluorescence intensity at the tumor site, indicating the EPR effect of both nanoparticle types (**Figure** **4**A). After 24 h, the mice were euthanized, and their major organs and tumors were harvested (Figure 4B,C). Mice treated with HER2‐Cy5.5‐ILs exhibited the highest accumulation of Cy5.5, ≈1.5‐ and 7.8‐fold higher than in mice treated with Cy5.5‐Lipo and free Cy5.5, respectively. Collectively, these findings suggest that ILs may be more efficiently delivered to tumor sites via increased retention and endocytosis by trastuzumab modification and passive targeting through the EPR effect.

### 2.5 In Vivo Antitumor Efficacy of HER2‐TNM A‐ILs

**Figure 5:**
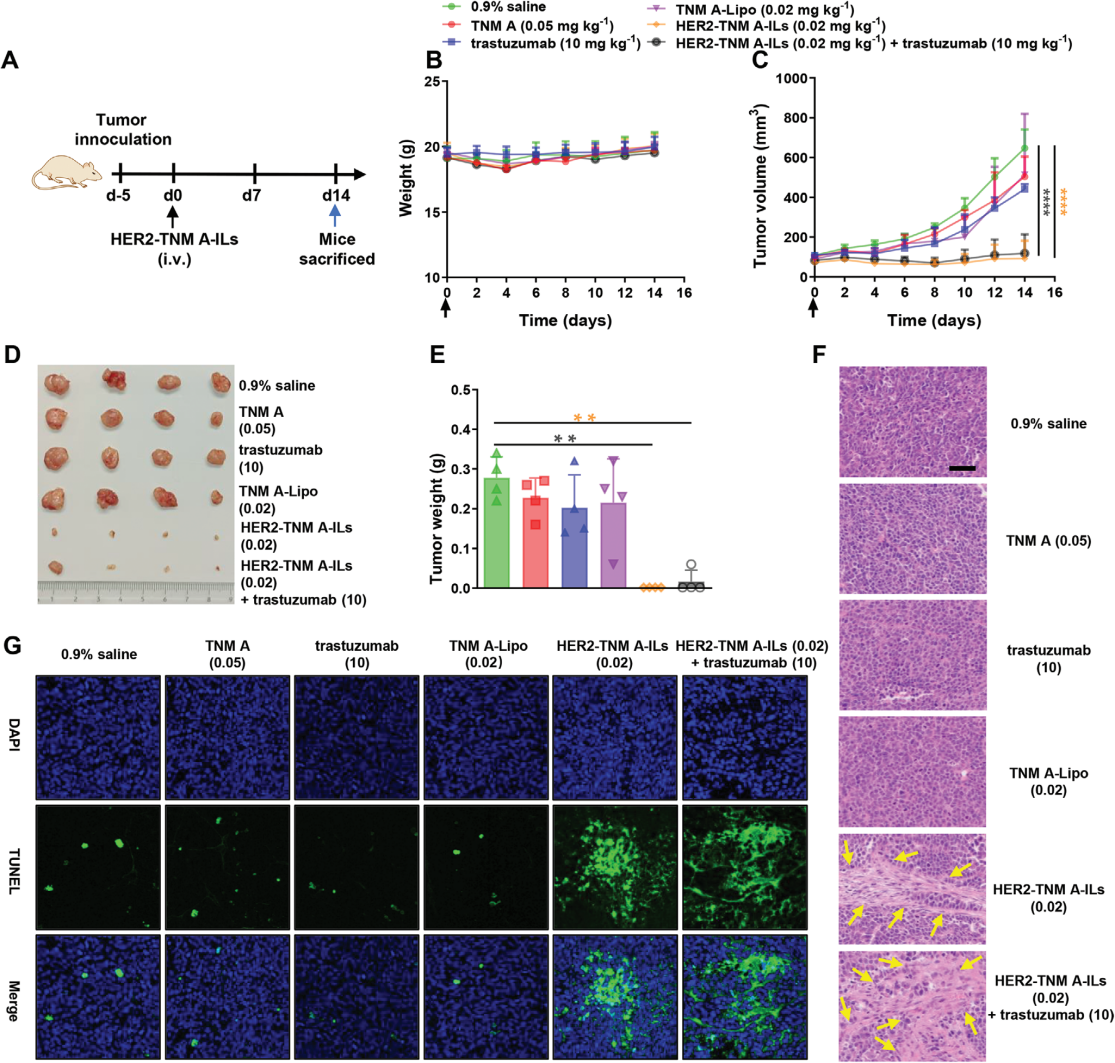
In vivo antitumor effects of free TNM A, TNM A‐Lipo, and HER2‐TNM A‐ILs in BALB/c nude mice bearing KPL‐4 tumors. A) Schematic illustration of respective drug treatment, as represented by HER2‐TNM A‐ILs. B) Day 14 body weights of treated mice (*n* = 5). C) Tumor volumes of treated mice (*n* = 5). D) Photographs of representative tumor blocks on day 14. E) Day 14 tumor weights of treated mice (*n* = 4). F) H&E staining of tumor tissue of the treated mice (40×). Scale bars: 50 µm. G) TUNEL staining (green) of tumors in the treated mice (40×). Data are expressed as the mean ± SD. Unpaired t‐tests (C) and One‐way ANOVA (E), (*) < 0.05, (**) < 0.01, (***) < 0.001, and (****) < 0.0001. H&E, hematoxylin and eosin; HER2‐TNM A‐ILs, anti‐human epidermal growth factor receptor 2 (trastuzumab)‐coated tiancimycin A‐loaded immunoliposomes; IL, immunoliposomes; TNM A, tiancimycin A.

To assess the safety of intravenously administered HER2‐TNM A‐ILs and TNM A‐Lipo nanoparticles, we performed a hemolysis assay. Based on the results of the red blood cell hemolysis assay, neither TNM A‐Lipo nor HER2‐TNM A‐ILs exhibited hemolytic toxicity within the concentration range of 0.001–10,000 nm (Figure S8, Supporting Information). Using a subcutaneous KPL‐4 tumor model, we next evaluated the therapeutic efficacies of intravenously administered HER2‐TNM A‐ILs and TNM A‐Lipo. Female tumor‐bearing BALB/c nude mice (*n* = 5) were administered HER2‐TNM A‐ILs (0.02 mg kg^−1^), TNM A‐Lipo (0.02 mg kg^−1^), and HER2‐TNM A‐ILs (0.02 mg kg^−1^) in combination with trastuzumab (10 mg kg^−1^); the control mice received trastuzumab (10 mg kg^−1^) and free TNM A (0.05 mg kg^−1^) (**Figure** **5**). Importantly, no significant weight loss was observed in any treatment group, suggesting that these treatment regimens were well tolerated (Figure 5B; Figure S9, Supporting Information). However, a single dose of free TNM A, TNM A‐Lipo, or trastuzumab only marginally inhibited tumor growth. Conversely, a single low dose of HER2‐TNM A‐ILs or a combination of HER2‐TNM A‐ILs and trastuzumab could significantly suppress tumor growth (Figure 5C; Figure S9, Supporting Information). Mice treated with HER2‐TNM A‐ILs exhibited the smallest tumor masses, confirming the superior antitumor effects of HER2‐TNM A‐ILs (Figure 5D,E). Notably, HER2‐TNM A‐ILs exerted substantially stronger antitumor activity than trastuzumab, which only slightly inhibited tumor growth following a single administration. Histological examination using hematoxylin and eosin (H&E)‐stained sections revealed extensive areas of necrosis in the tumors of mice treated with HER2‐TNM A‐ILs or HER2‐TNM A‐ILs in combination with trastuzumab. H&E‐stained tumor tissues of both groups displayed prominent tumor cell shrinkage and disrupted cell membranes, significantly more than that observed in the TNM A‐Lipo and free TNM A groups at the same dosage (Figure 5F). Consistent with the H&E staining findings, terminal deoxynucleotidyl transferase (TdT)‐mediated dUTP nick‐end labeling (TUNEL) staining demonstrated a higher level of KPL‐4 cell apoptosis in the HER2‐TNM A‐IL and HER2‐TNM A‐ILs + trastuzumab groups than that in the other treatment groups (Figure 5G).

In the same KPL‐4 mouse model, a single dose of HER2‐TNM A‐ILs (0.02 mg kg^−1^) afforded a more pronounced antitumor effect than two doses of cyclic arginine‐glycine‐aspartate (cRGD)‐functionalized liposomes (cRGD‐Lip‐TNM A; 0.02 mg kg^−1^) as previously reported.^[^ ^19b^ ^]^ Treatment with ILs nearly stopped the KPL‐4 tumor growth, whereas treatment with the α_v_β_3_‐integrin receptor‐targeting liposomes only reduced two‐thirds of the tumor mass; this difference could be attributed to the more efficient targeting of trastuzumab monoclonal antibodies than that of cRGD peptides, along with the HER2‐facilitated endocytosis of ILs when compared with α_v_β_3_‐integrin receptors. Furthermore, a single administration of HER2‐TNM A‐ILs (0.02 mg kg^−1^) elicited a pronounced antitumor effect when compared with MM‐302 ILs, administered once every 7 days at 3 mg kg^−1^ for three doses.^[^ ^12a^ ^]^ The enhanced potency of the encapsulated enediyne TNM A compared with that of doxorubicin in MM‐302 may be a key contributing factor. Additionally, whether HER2‐TNM A‐ILs exhibit bystander effects, as reported for uncialamycin‐ADCs, in small‐cell lung cancer mouse models needs to be explored in the future.^[^ ^24a^ ^]^ These results underscore the need for further research into the use of anthraquinone‐fused enediynes as payloads for developing potent targeted anticancer therapeutics.

### 2.6 In Vivo Toxicity of HER2‐TNM A‐ILs

**Figure 6:**
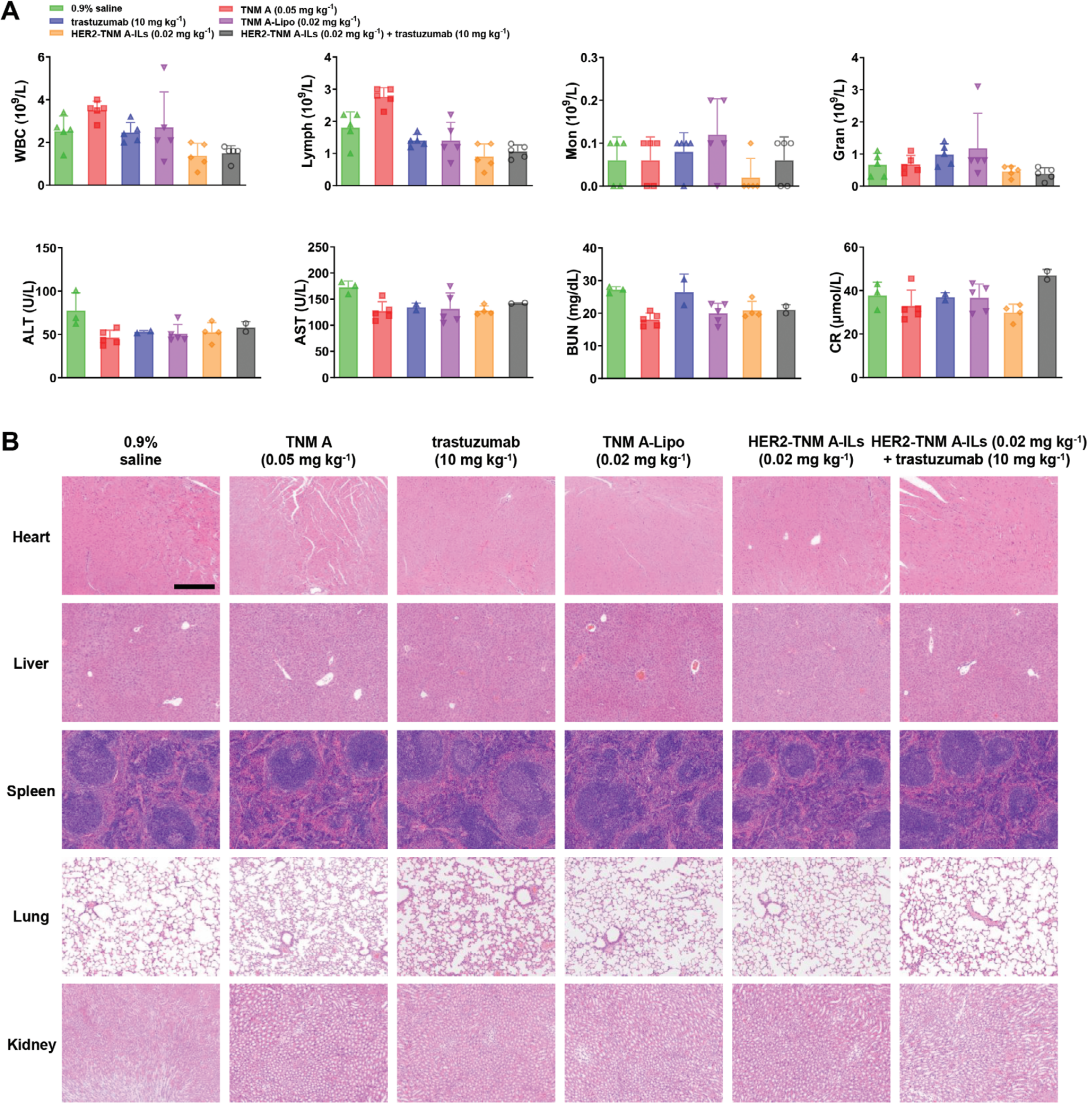
Blood analyses of mice and H&E‐stained tissue sections of major organs of treated mice. A) Blood analyses of mice treated with free TNM A, TNM A‐Lipo, and HER2‐TNM A‐ILs in BALB/c nude mice bearing KPL‐4 tumors (*n* = 5). Blood samples were collected from different treatment groups on day 14. B) H&E staining of the major organs of treated mice. Heart, liver, spleen, lung, and kidney (10 ×). The scale bar represents 200 µm. Data are expressed as the mean ± SD. H&E, hematoxylin and eosin; HER2‐TNM A‐ILs, anti‐human epidermal growth factor receptor 2 (trastuzumab)‐coated tiancimycin A‐loaded immunoliposomes; IL, immunoliposomes; TNM A, tiancimycin A.

To evaluate the in vivo toxicity of the aforementioned treatments, we performed biochemistry assays using serum derived from experimental mice. HER2‐TNM A‐IL‐treated mice did not exhibit increased white blood cell, lymphocyte (lymph), monocyte (Mon), or granulocyte (Gran) counts when compared with 0.9% saline‐treated mice. This absence of altered blood parameters indicates that there was no detectable inflammatory response (**Figure** **6**A).

Similarly, the levels of hepatic functional markers, including aspartate aminotransferase (AST) and alanine transaminase, were comparable between HER2‐TNM A‐ILs‐treated mice and 0.9% saline‐treated mice. This finding suggests that HER2‐TNM A‐ILs did not adversely affect liver function in treated mice. Additionally, the levels of two renal functional markers, blood urea nitrogen and serum creatinine, were within normal ranges, thereby suggesting that HER2‐TNM A‐ILs did not cause kidney dysfunction in mice. Pathological examination of the heart, liver, lungs, spleen, and kidneys of treated mice revealed no significant damage at the administered dose (Figure 6B). Taken together, these preliminary findings demonstrate that HER2‐TNM A‐ILs lack evident toxicity in vivo. This aligns with our prior report, which found no obvious toxicity when TNM A‐encapsulated liposomes were administered at a dosage of 0.02 mg kg^−1^.^[^ ^19b^ ^]^ The excellent targeting ability of trastuzumab, in combination with the rapid and complete cytotoxic activity of TNM A, allows HER2‐TNM A‐ILs to elicit a potent antitumor effect at a very low dosage of 0.02 mg kg^−1^. Both high selectivity and low‐dose administration of HER2‐TNM A‐ILs substantially enhance the safety of designed ILs.

## 3 Conclusion

HER2‐targeted therapeutics, including trastuzumab and T‐DXd, have revolutionized the treatment of HER2‐positive tumors.^[^ ^27^ ^]^ In the current study, we designed, synthesized, and characterized a novel formulation composed of trastuzumab‐decorated immunoliposomes (HER2‐TNM A‐ILs) that exhibited remarkable efficacy in suppressing KPL‐4 tumor growth in mice treated with a single dose without systemic toxicity (Figures 5 and 6). To the best of our knowledge, our study, for the first time, prepared ILs encapsulating a small quantity of the highly potent cytotoxin TNM A, showing superior selectivity against HER2‐positive breast cancer cells at single‐digit nanomolar concentrations (Figure 2). Engagement of HER2 by these ILs was further confirmed using fluorescent surrogates, including Cou‐6 and Cy5.5, in experiments involving KPL‐4 and SKBR3 cells or KPL‐4 tumor‐bearing mice (Figures 3 and 4). The superior antitumor activity of HER2‐TNM A‐ILs can be attributed to the synergistic effect of trastuzumab and TNM A, which exhibit strong DNA‐damaging activity. Notably, the specific delivery of these ILs to HER2‐positive tumors mediated by trastuzumab further enhanced the antitumor effects.^[^ ^28^, ^29^ ^]^

To date, three natural enediyne products–neocarzinostatin, C‐1027, and calicheamicin–have been utilized in cancer therapy. In recent decades, there has been rapid progress in the discovery and engineering of new anthraquinone‐fused enediynes such as TNM A.^[^ ^19a^ ^]^ However, their substantial cytotoxicity poses considerable challenges in harnessing their potent antitumor activity. With the advancement of liposome‐based technology and the adaptable use of commercial monoclonal antibodies such as trastuzumab in the form of ILs, a promising avenue has emerged for translating these novel enediynes for cancer therapy. Despite the early development of ILs in the last century,^[^ ^11^, ^12^, ^15^ ^]^ to the best of our knowledge, no ILs have been approved for treating human cancers. Only a relatively small percentage of the ILs likely reach the tumor sites via the EPR effect (Figure 4). Although HER2‐TNM A‐ILs have demonstrated excellent antitumor activity, further enhancing the accumulation of these ILs at relevant tumor sites is crucial.^[^ ^31^ ^]^ Additionally, there is considerable potential for combination therapies involving HER2‐TNM A‐ILs and other anticancer drugs to achieve improved therapeutic outcomes and reduced drug resistance.

Herein, we prepared ILs with cores encapsulating ≈190 TNM A molecules and modified 830 trastuzumab. Conversely, MM‐302 comprises ≈20000 molecules of doxorubicin encapsulated in its core and 45 single‐chain anti‐HER2 antibodies conjugated to its surface. Functionalization of liposome surfaces with antibodies may enhance the accumulation of nanomedicines in tumors via the antibody‐mediated recognition of specific antigens overexpressed on tumor cell membranes. However, the coupling of whole antibodies leads to high immunogenicity and reduces the average residence time of ILs in the bloodstream. The development of monoclonal antibody engineering facilitates the use of monoclonal antibody fragments capable of maintaining antigen‐binding properties, such as Fab or single‐chain fragment variable fragments, which may be used for the future engineering of TNM A‐based ILs. In addition to monoligand‐modified ILs, the potential of bispecific ILs targeting different receptors expressed on the tumor cell surface needs to be explored.

## 4 Experimental Section

### Materials

TNM A was prepared according to the previous reports.^[^ ^19b^ ^]^ PC, cholesterol, and DSPE‐PEG_2000_ were purchased from Sigma‐Aldrich (Munich, Germany) or Huateng Pharmaceutical (Changsha, China). DSPE‐PEG_2000_‐NHS was purchased from Ruixi Biotechnology (Shanxi, China). Trastuzumab was purchased from Roche (Basel, Switzerland). Dialysis bags were purchased from Yuanye Biotechnology (Shanghai, China). Methanol, ethanol, and dichloromethane were purchased from Sinopharm Chemical Reagent Co., Ltd. (Shanghai, China). Cell Counting Kit‐8 was purchased from Dongren Chemical Technology Co., Ltd. (Kami Kumamoto, Japan). DMEM and RPMI 1640 media, as well as fetal bovine serum (FBS) and 0.25% (w/v) trypsin solution were purchased from Gibco (Grand Island, NY, USA). Penicillin and streptomycin were purchased from HyClone (Logan, UT, USA). Phosphate buffered saline (PBS) (pH 7.4) was from Gibco (Paiseley, UK).

### Cell Lines and Culture Conditions

KPL‐4, SKBR3, MCF‐7, and NCM‐460 cells were from the cell bank of the Institute of Cellular Biology of Chinese Academy of Sciences (Beijing, China). MCF‐7 cells and NCM‐460 cells were cultured in modified RPMI 1640 medium containing 10% (v/v) FBS and 1% penicillin‐streptomycin. KPL‐4 cells and SKBR3 cells were cultured in DMEM medium containing 10% (v/v) FBS and 1% penicillin‐streptomycin.

### Animals

Female BALB/c nude mice (6 weeks) weighing 20 ± 2 g each were purchased from Hunan Slack Jingda Experimental Animal Co., Ltd. (Changsha, China). All mice were kept in a controlled environment on a 12 h light/dark cycle, with an ambient temperature of 26 °C and relative humidity of 50%. All animal experiments were approved by the Animal Ethics Committee of Central South University (no. 2019sydw0178).

### Synthesis of TNM A‐Lipo and NHS‐Lipo

TNM A‐Lipo and NHS‐Lipo were prepared using the ethanol evaporation method. To prepare TNM A‐Lipo, PC, cholesterol, DSPE‐PEG_2000_, and TNM A were first dissolved at a molar ratio of 56/39/5/0.2 in ethanol (2 mL), and then poured into pre‐warmed PBS (55 °C, 5 mL). The mixture was vigorously stirred at 55 °C for 2 h, then the desired liposomes were obtained after removal of ethanol under vacuum. NHS‐Lipo were similarly prepared using PC, cholesterol, DSPE‐PEG_2000_, DSPE‐PEG_2000_‐NHS, and TNM A at a molar ratio of 56/39/5/1/(0.004–0.6).

### Preparation and Characterization of HER2‐TNM A‐ILs

Trastuzumab (10 mg mL^−1^, 5 mL) was added to a dialysis bag (MWCO: 100 KDa) and sealed, which was then dialyzed in the dialysate (1 L, 50 mm Na_2_HPO_4_, 50 mm NaH_2_PO_4_, 10 mm EDTA, 0.15 m NaCl, pH 7.4) at 4 °C for 6 h. The dialysate was changed every 2 h. After dialysis, the resulting antibody (10 mg mL^−1^, 0.29 mL) was mixed with NHS‐Lip (PBS, pH 7.4) at a molar ratio of 1:50 (5 mL) and incubated at room temperature with gentle shaking for 3 h. The resulting mixture was added to a dialysis bag (MWCO: 300 KDa) and placed in a PBS solution to remove the trastuzumab that was not linked to the liposomes. The dynamic sizes and zeta potentials of TNM A‐Lipo and HER2‐TNM A‐ILs were determined by Zetasizer Nano 90 (Malvern). TNM A‐Lipo and HER2‐TNM A‐ILs solution was dropped onto the copper net covered with carbon film for 30 s, respectively. Then the excess liquid was removed with filter paper and the copper net was dried for ≈3 min under the light. The morphologies of the TNM A‐Lipo and HER2‐TNM A‐ILs were characterized using the transmission electron microscope (TEM) (Tecnai G2 20S‐Twin).

The concentration of immunoliposome particles was analyzed using a NanoFCM nanoflow cytometer.^[^ ^32^ ^]^ The total number of TNM A drug molecules within the nano‐formulation system was calculated based on the amount of TNM A administered and the encapsulation rate. The total number of antibody molecules within the nanopreparation system was calculated based on the input dose of trastuzumab and the linkage rate. Finally, the average number of TNM A encapsulated in one nanoparticle and how much trastuzumab was modified was estimated based on the total number of nanoparticles within the system. The content of TNM A loaded was determined via high‐performance liquid chromatography (HPLC). TNM A was detected with the Waters E2695 HPLC system equipped with a photo‐diode array detector and a Welch AQ‐C18 column (5 µm, 250 × 4.6 mm, Welch Materials Inc., Shanghai, China). The mobile phase consisted of buffer A (ultrapure H_2_O containing 0.1% HCO_2_H) and buffer B (chromatographic grade CH_3_CN containing 0.1% HCO_2_H) at a flow rate of 1 mL min^−1^. A gradient program (90% buffer A from 0 to 1 min; 90% buffer A to 5% buffer A from 1 to 12 min; 5% buffer A from 12 to 14 min; 5% buffer A to 90% buffer A from 14 to 20 min; 90% buffer A from 20 to 22 min) was applied to analyze TNM A at 540 nm. The TNM A amount was quantified based on the calibration curve with the authentic TNM A standard.

### Antibody Concentration Determination and SDS‐PAGE Analysis

Antibody concentrations were measured by bicinchoninic acid protein assay reagent. The protein standards (1 mg mL^−1^) were diluted with PBS into different concentrations of bovine serum albumin at 1, 0.8, 0.6, 0.4, 0.2, 0.1, 0.05, and 0 mg mL^−1^. The total volume of standards and PBS was 20 µL per well. The different concentrations of standards were added to the 96‐well cell culture plate. The sample wells were filled with 19 µL of PBS and 1 µL of the sample to be tested in a total volume of 20 µL. The absorbance at 562 nm was measured using a microplate reader (TECAN). The protein concentration of the sample was calculated from the standard curve and absorbance. Antibody integrity and molecular weight of ILs were assessed by polyacrylamide gel electrophoresis.

### In Vitro Drug Release

Free TNM A, TNM A‐Lipo and HER2‐TNM A‐ILs (1 mL) in a dialysis bag with a molecular retention of 3500 MWCO was dialyzed in 30 mL PBS (pH 7.4, 10 mm) with 0.5% sodium dodecyl sulfate (SDS) (200 rpm, 37 °C ± 0.5 °C). The PBS solutions (1 mL) containing the released TNM A at 0, 1, 4, 8, 12, 24, 36, 48, 60, 72, 84, 96, 108, 120, 132, and 144 h were used to analyze TNM A using HPLC. An equal amount of PBS was added to the dialysis systems each time. The cumulative release rate of TNM A was then calculated based on the peak area from the HPLC analysis.

### In Vitro Cytotoxicity Assay

A CCK‐8 assay was used to measure the inhibition rates of TNM A, TNM A‐Lipo, and HER2‐TNM A‐ILs. KPL‐4, SKBR3, MCF‐7, and NCM‐460 cells (5 × 10^3^ cells in 100 µL) were seeded in 96‐well plates and allowed to settle overnight. These cells were then treated with 0.0001, 0.01, 0.1, 0.5, 1, 5, 50, or 100 nm of TNM A, TNM A‐Lipo, and HER2‐TNM A‐ILs for 8 or 48 h, and cells were treated with blank HER2‐ILs at varying concentrations of 0.01−1.0 mg mL^−1^ for 48 h. After incubation, the CCK‐8 solution (10 µL) was added to each well. The cells were incubated for an additional hour at 37 °C, and the optical density (OD) was measured with a microplate reader (TECAN) at a wavelength of 450 nm. The cell viability could be calculated according to formula:

(1) $$\mathrm{Cell}\;\mathrm{inhibition}\;\mathrm{rate}\;\%\;=\;\frac{A_{\mathrm{sample}}-A_{\mathrm{blank}}}{A_{\mathrm{control}\;}-A_{\mathrm{blank}}}\;\times \;100\%$$

### Cellular Uptake

KPL‐4 and SKBR3 cells were seeded in 6‐well plates at 20 × 10^4^ cells well^−1^ and incubated for 24 h before treatment. Cou‐6, Cou‐6‐Lipo and HER2‐Cou‐6‐ILs (100 or 200 ng well^−1^) were then added. Alternatively, the cells were preincubated with 10 µM trastuzumab for 2 h. Cou‐6, Cou‐6‐Lipo, and HER2‐Cou‐6‐ILs (100 or 200 ng well^−1^) were then added. Ice‐cold PBS was used to remove the remaining nanoparticles. The cells were next fixed in 4% paraformaldehyde, and 1 µg mL^−1^ DAPI was added for nuclear staining. Cellular uptake of the nanoparticles was observed using inversion fluorescence microscopy (Leica DFC7000T) and quantified using the Photoshop CS6 software. Cou‐6, Cou‐6‐Lipo, and HER2‐Cou‐6‐ILs (100 or 200 ng well^−1^) were incubated with KPL‐4 cells at 37 °C for 2 h. Cells were digested with trypsin and then centrifuged. The resulting supernatant was aspirated and 0.5 mL of PBS was added. Fluorescence detection was performed using BD flow cytometer.

### Hemolysis Assay

The hemolytic activity of TNM A‐Lipo, and HER2‐TNM A‐ILs were evaluated. TNM A‐Lipo, and HER2‐TNM A‐ILs dissolved in 0.9% saline at various concentrations were incubated with red blood cells in a suspension at 37 °C for 2 h and then centrifuged at 3000 rpm for 5 min. Triton X‐100 and 0.9% saline solution were used as positive controls (100% lysis) and negative (0% lysis). The absorbance of the supernatant was measured at 570 nm. The hemolysis rate was calculated using the following equation:

(2) $$\mathrm{Hemolysis}\;\mathrm{rate}\;\%\;=\;\frac{A_{\mathrm{sample}}-A_{\mathrm{negative}}}{A_{\mathrm{positive}\;}-A_{\mathrm{negative}}}\;\times \;100\%$$

### In Vivo Imaging of KPL‐4 Human Breast Tumor Model

Subcutaneous KPL‐4 human breast tumor‐bearing mice were obtained by injecting KPL‐4 cells (5 × 10^6^ cells) in the right armpit of the mice. When the tumor volume of each BALB/c nude mouse reached to a value of ≈300–500 mm^3^, 200 µL of Cy5.5, Cy5.5‐Lipo, HER2‐Cy5.5‐ILs were injected into the tumor‐bearing mice via the tail vein at the Cy5.5 dosage of 1 mg kg^−1^. At different time points (2, 4, 24 h) post injection, the mice were anesthetized with isoflurane, and the distribution of the fluorescence in mice was determined using a near‐infrared fluorescence imaging system (PerkinElmer/IVIS Spectrum BL). At 24 h, the main organs and tumors were collected and their fluorescence images were acquired.

### In Vivo Antitumor Efficacy

The BALB/c nude mice were subcutaneously injected in the right armpit with KPL‐4 cells (5 × 10^6^ cells in 50 µL PBS and 50 µL Matrigel [BD Biocoat]) to establish the subcutaneous tumor‐bearing model. When the tumor volume of each BALB/c nude mouse reached ≈100 mm^3^, the mice were randomly divided into six groups (*n* = 5 mice per group): untreated control (0.9% saline), positive control trastuzumab (10 mg kg^−1^), free TNM A (0.05 mg kg^−1^), TNM A‐Lipo (0.02 mg kg^−1^), HER2‐TNM A‐ILs (0.02 mg kg^−1^) and positive control trastuzumab (10 mg kg^−1^) + HER2‐TNM A‐ILs (0.02 mg kg^−1^). One single dose of all groups was administered through the caudal vein. Tumor length (a) and width (b) were measured daily from the time of administration. Tumor volume (V) was calculated as V = 1/2 ab^2^. On the last day of the experiments, all BALB/c nude mice were euthanized, and their tumor, heart, liver, spleen, lung, and kidney were collected and fixed in 4% paraformaldehyde for hematoxylin and eosin (H&E) staining analysis. In addition, to investigate the apoptosis of tumor cells induced by TNM A, tumor tissues were further stained with terminal deoxynucleotidyl transferase‐mediated dUTP nick‐end labeling (TUNEL).

### Statistical Analysis

All data were presented as mean ± SD. Statistical analysis was presented using the GraphPad Prism 9.0 (GraphPad Software, USA). Shapiro–Wilk test was used to test for normal distribution. Student's t‐tests and one‐way ANOVA were used for the significant difference between groups. ^*^ *P* < 0.05 was significant difference, ^**^ *P* < 0.01 and ^***^ *P* < 0.001 were highly significant difference, ns, no significant difference.

## Conflict of Interest

The authors declare no conflict of interest.

## Supporting information

Supporting Information

## Data Availability

The data that support the findings of this study are available in the supplementary material of this article.
