# Supplementary material for: Anti‐HER2 Immunoliposomes: Antitumor Efficacy Attributable to Targeted Delivery of Anthraquinone‐Fused Enediyne
Source: Adv Sci (Weinh). 2024 Feb 14;11(17):2307865. doi: 10.1002/advs.202307865 (PMC11077693; doi:10.1002/advs.202307865)

ADVANCED SCIENCE
Open Access

Supporting Information

for *Adv. Sci.*, DOI 10.1002/advs.202307865

Anti-HER2 Immunoliposomes: Antitumor Efficacy Attributable to Targeted Delivery of Anthraquinone-Fused Enediyne

Xueqiong Feng, Zhongqing Wen, Xiangcheng Zhu, Xiaohui Yan, Yanwen Duan and Yong Huang**

Supporting Information

Anti-HER2 Immunoliposomes: Antitumor Efficacy Attributable to Targeted Delivery of Anthraquinone-Fused Enediyne

Xueqiong Feng, Zhongqing Wen, Xiangcheng Zhu, Xiaohui Yan, Yanwen Duan and Yong Huang**

X. Q. Feng, Z. Q Wen, Dr. X. C. Zhu, Prof. Y. W. Duan, Prof. Y. H
Xiangya International Academy of Translational Medicine, Central South University, Changsha, Hunan 410013, China
E-mail: ywduan66@sina.com; jonghuang@ihm.ac.cn

Prof. Y. Huang
Institute of Health and Medicine, Hefei Comprehensive National Science Center, Hefei，Anhui 230093, China
E-mail: jonghuang@ihm.ac.cn

Dr. X. C. Zhu, Prof. Y. W. Duan
Hunan Engineering Research Center of Combinatorial Biosynthesis and Natural Product Drug Discover, Changsha, Hunan 410011, China

Prof. X. H. Yan
State Key Laboratory of Component-based Chinese Medicine, Tianjin University of Traditional Chinese Medicine, Tianjin, 301617, China

Prof. Y. W. Duan, Prof. Y. Huang
National Engineering Research Center of Combinatorial Biosynthesis for Drug Discovery, Changsha, Hunan 410011, China

Table of contents

Figure S1. Characterization of different nanoparticles... 3

Figure S2. TNM A released from TNM A-Lipo and HER2-TNM A-ILs... 4

Figure S3. Particle concentration measurement of HER2-TNM A-ILs and HER2-Cou-6-ILs by NanoFCM... 5

Figure S4. The cytotoxicity of blank HER2-ILs against SKBR3 and MCF-7 cells.... 6

Figure S5. In vitro cytotoxicities of TNM A, TNM A-Lipo and HER2-TNM A-ILs to SKBR3 cells and MCF-7 cells...7

Figure S6. The hydrodynamic size of Cou-6-Lipo and HER2-Cou-6-ILs by DLS.... 8

Figure S7. Intracellular uptake of Cou-6-Lipo and HER2-Cou-6-ILs as TNM A surrogates..... 9

Figure S8. In vitro hemolysis assay of TNM A-Lipo and HER2-TNM A-ILs....................10

Figure S9. In vivo antitumor effects of free TNM A, TNM A-Lipo, and HER2-TNM A-ILs in BALB/c nude mice bearing KPL-4 tumors.... 11

Figure S1. Characterization of different nanoparticles.

Material Composition	Molar Ratio	Size (nm)	PDI	Zeta-potential (mV)
PC / CHOL / DSPE-PEG$_{2000}$ / DSPE-PEG$_{2000}$-NHS / Trastuzumab / TNM A	56 : 39 : 5 : 0 : 0 : 0.2	202.8	0.245	-1.75
	56 : 39 : 5 : 1 : 0.02 : 0.004	186.3	0.225	-1.82
	56 : 39 : 5 : 1 : 0.02 : 0.02	182.8	0.270	-1.66
	56 : 39 : 5 : 1 : 0.02 : 0.1	189.0	0.238	-1.65
	56 : 39 : 5 : 1 : 0.02 : 0.2	185.6	0.217	-1.86
	56 : 39 : 5 : 1 : 0.02 : 0.6	199.5	0.269	-1.77

Figure S2. TNM A released from TNM A-Lipo and HER2-TNM A-ILs.

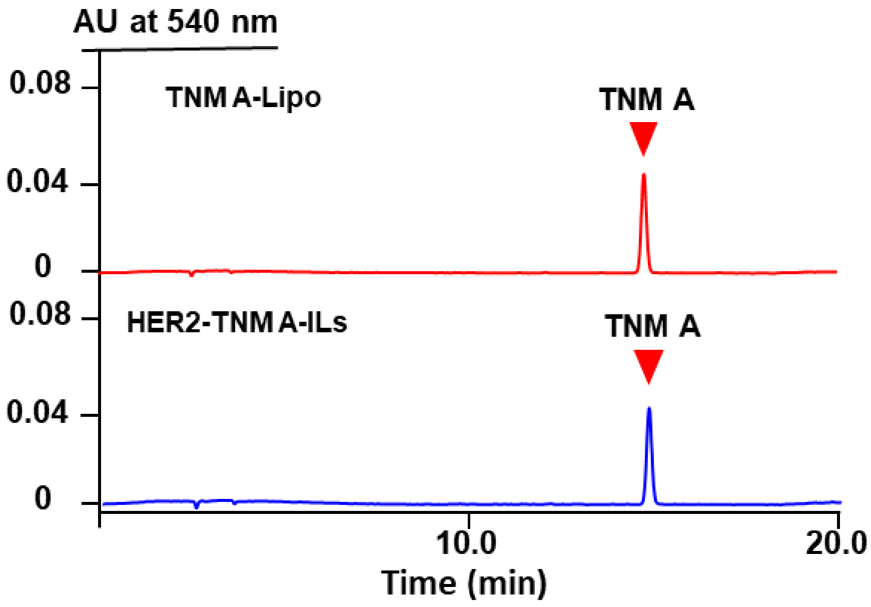

Figure S3. Particle concentration measurement of HER2-TNM A-ILs and HER2-Cou-6-ILs by NanoFCM.

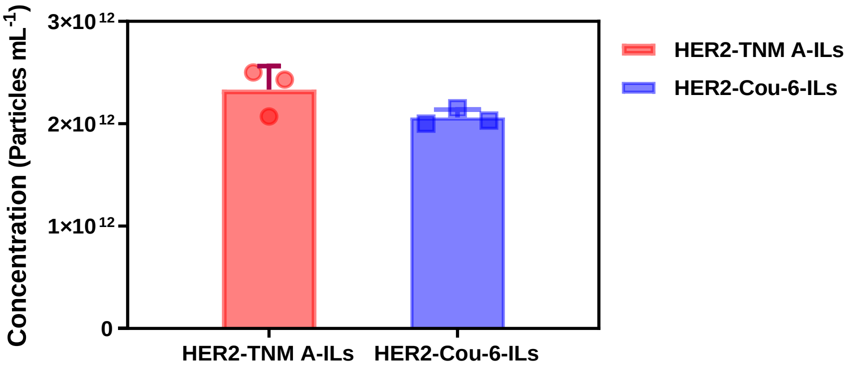

Figure S4. The cytotoxicity of blank HER2-ILs against SKBR3 and MCF-7 cells.

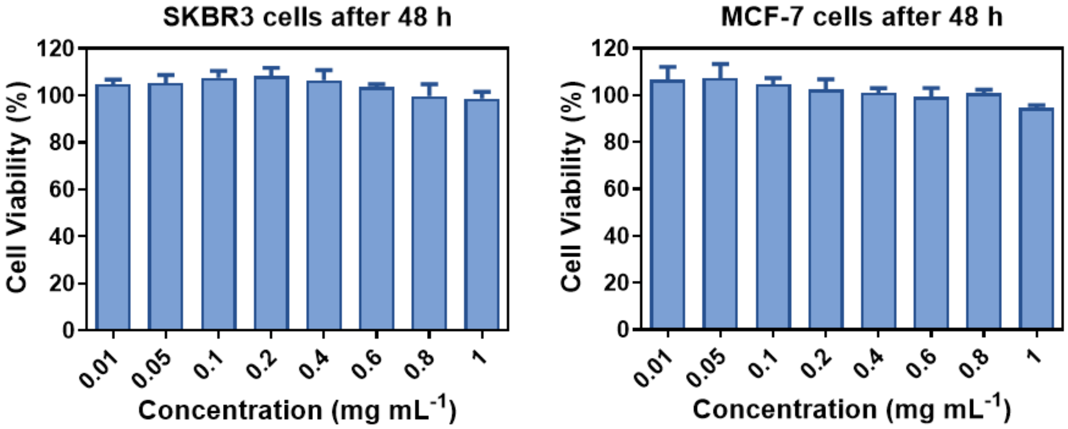

Figure S5. In vitro cytotoxicities of TNM A, TNM A-Lipo and HER2-TNM A-ILs to SKBR3 cells and MCF-7 cells.

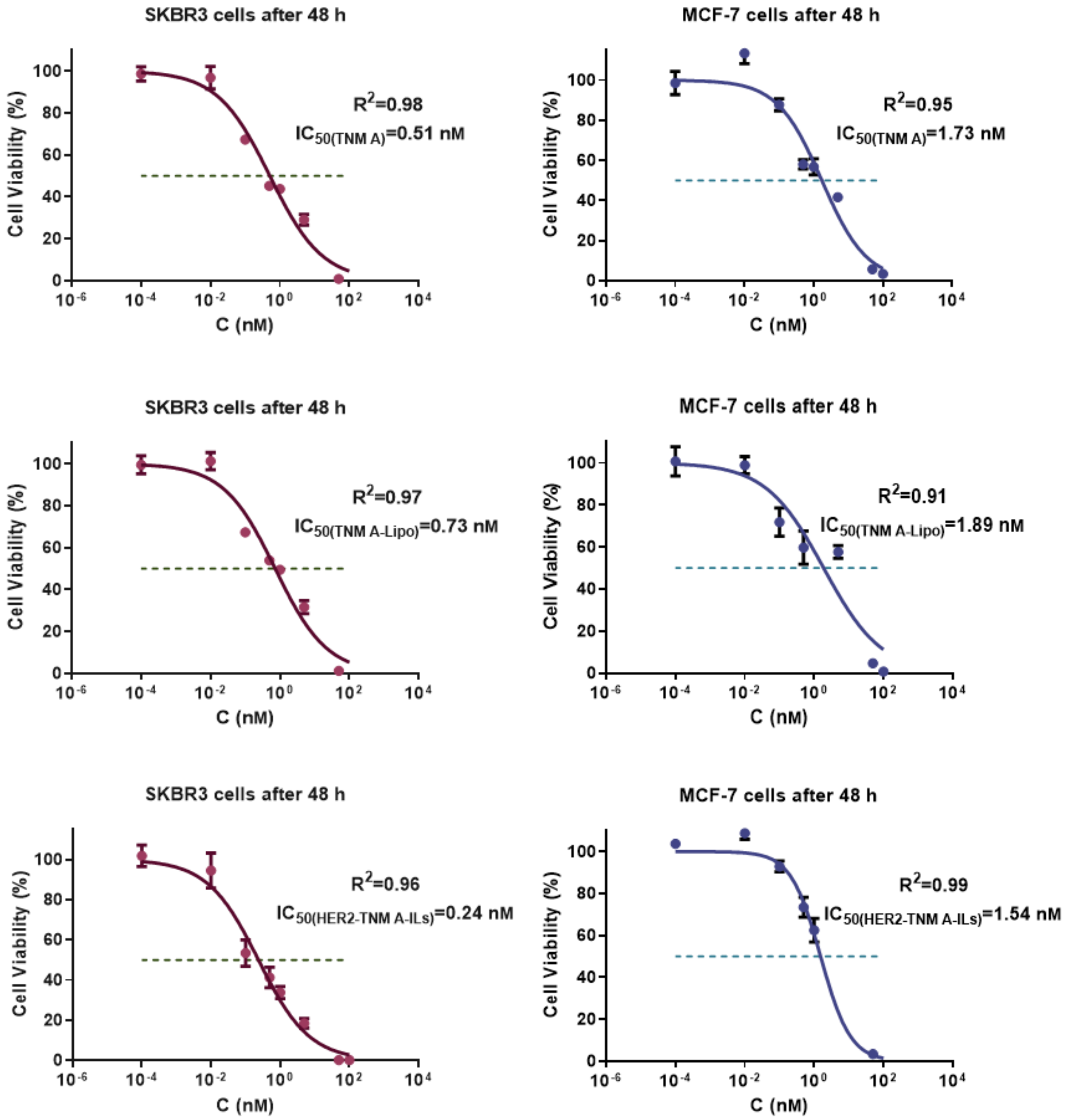

Figure S6. The hydrodynamic size of Cou-6-Lipo and HER2-Cou-6-ILs by DLS.

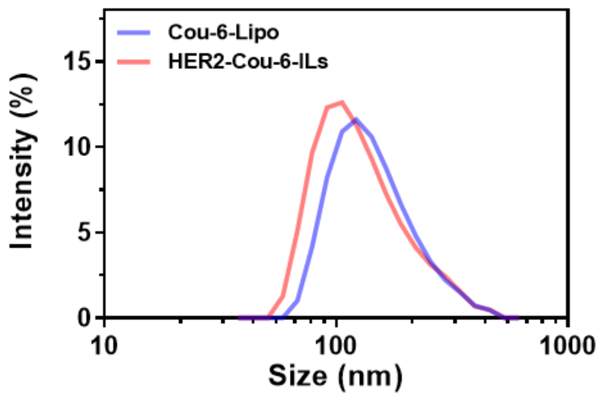

Figure S7. Intracellular uptake of Free Cou-6, Cou-6-Lipo and HER2-Cou-6-ILs as TNM A surrogates. Fluorescent images of intracellular uptake of the conjugates with or without the trastuzumab. SKBR3 cells were incubated with Free Cou-6, Cou-6-Lipo and HER2-Cou-6-ILs for 2 h, with or without preincubation with 10 μM trastuzumab for 2 h. Scale bar: 200 μm. Green: Cou-6; Blue: DAPI.

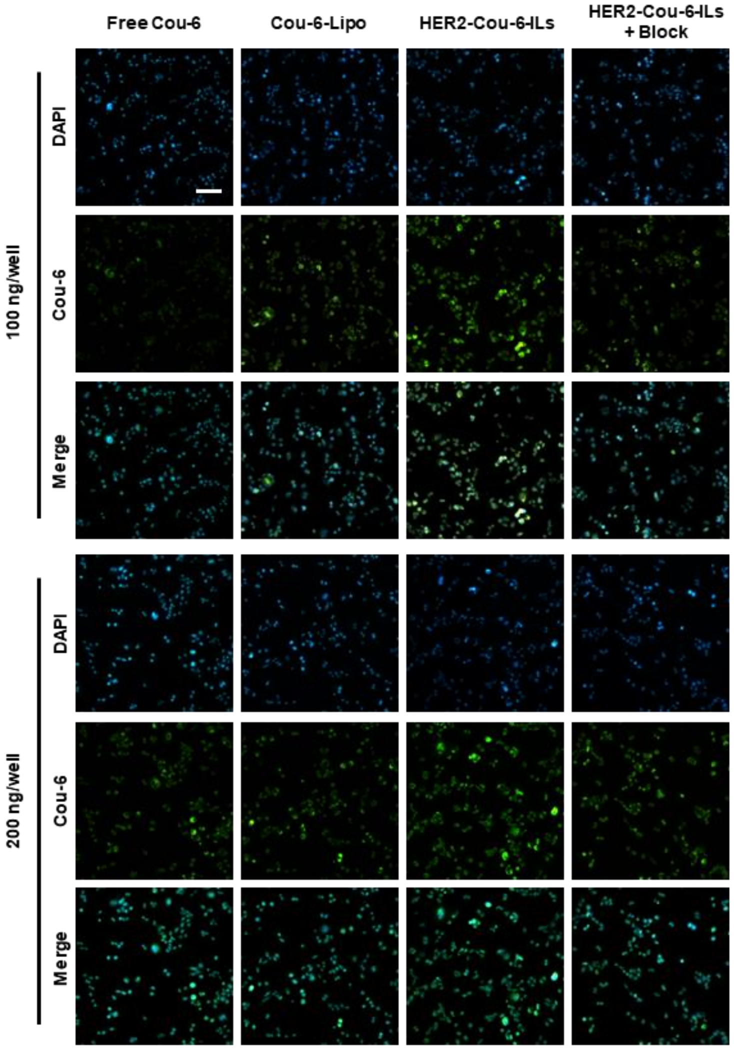

Figure S8. In vitro hemolysis assay of TNM A-Lipo and HER2-TNM A-ILs. (A) Hemolysis rate (%) of various concentration of TNM A-Lipo and HER2-TNM A-ILs (B) Images of red blood cells treated with TNM A-Lipo and HER2-TNM A-ILs.

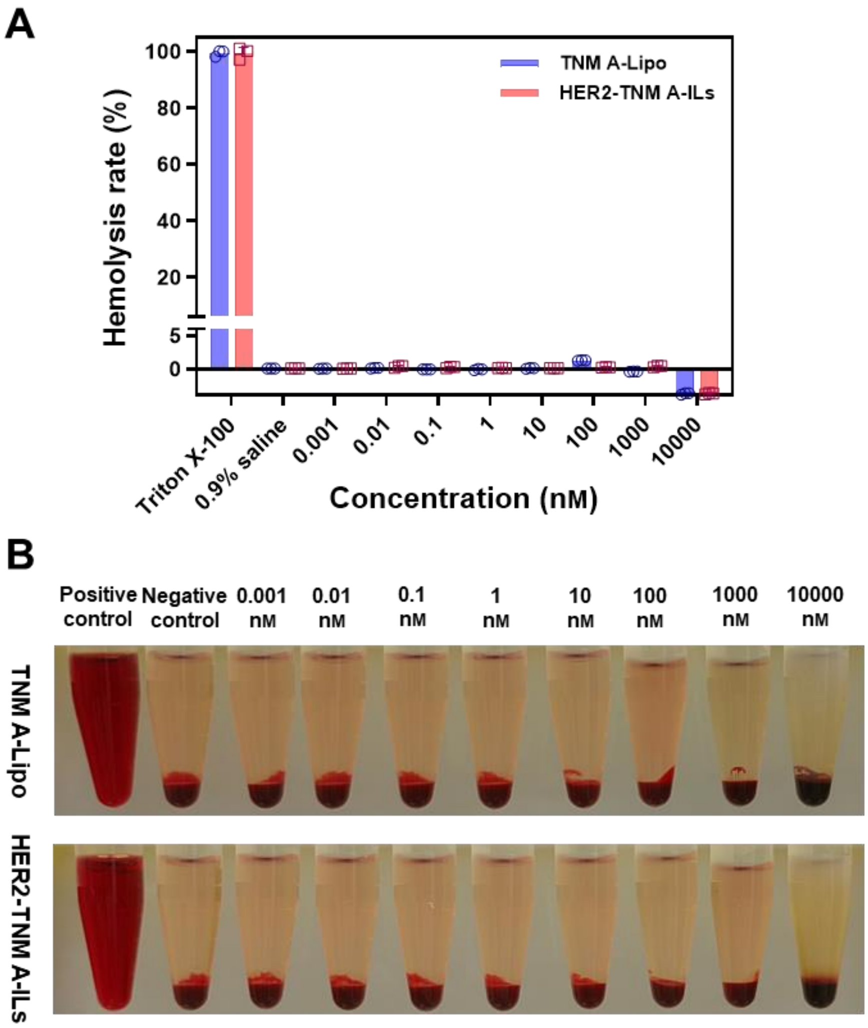

Figure S9. In vivo antitumor effects of free TNM A, TNM A-Lipo, and HER2-TNM A-ILs in BALB/c nude mice bearing KPL-4 tumors. (A) Relative weights of treated mice on day 14 (n = 5). (B) Relative tumor volumes of treated mice (n = 5).

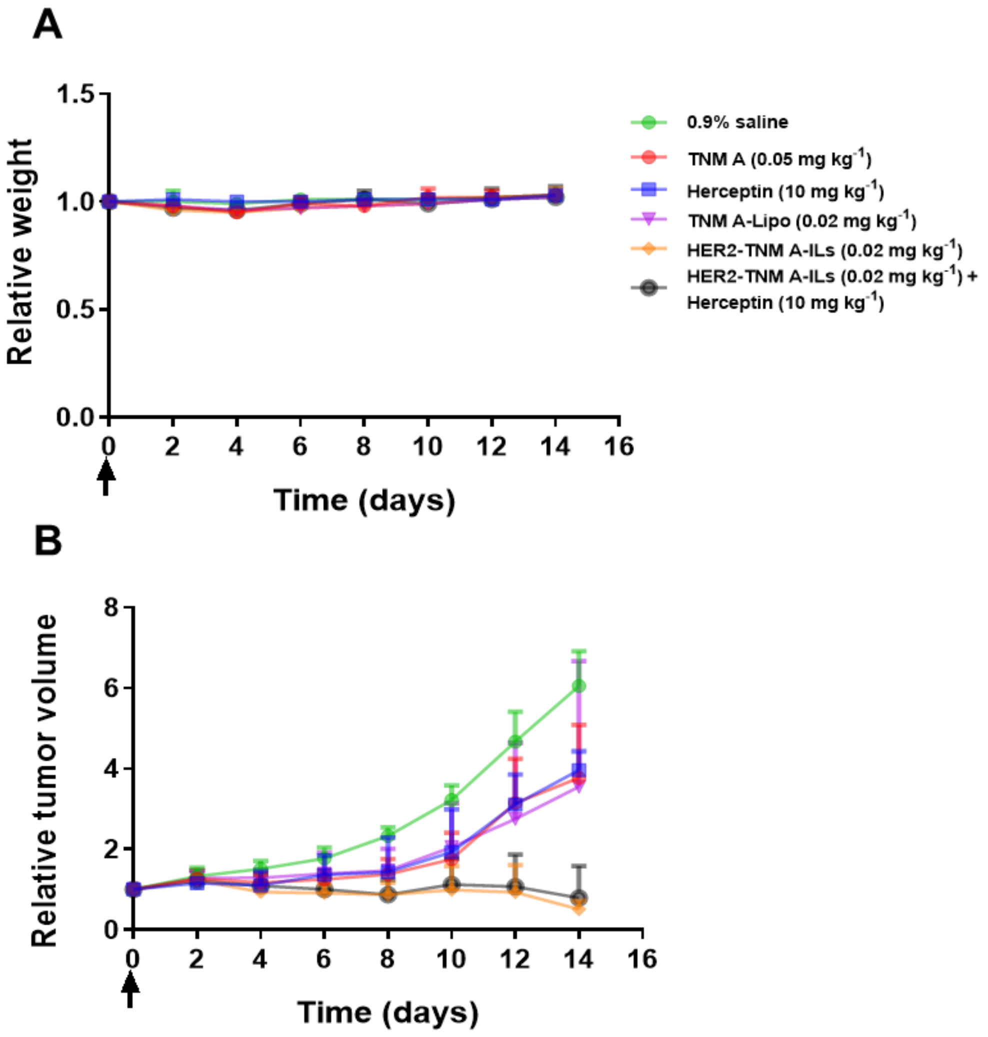

Supplement: Supplementary file 1 — Supporting Information [file ADVS-11-2307865-s001.pdf]
